# Retrospective Study on Prevalence, Specificity, Sex, and Age Distribution of Alloimmunization in Two General Hospitals in Athens

**DOI:** 10.4274/tjh.galenos.2020.2019.0459

**Published:** 2020-08-28

**Authors:** Marianna Politou, Serena Valsami, Georgios Dryllis, Maria Christodoulaki, Christina Cheropoulou, Abraham Pouliakis, Maria Baka, Konstantinos Stamoulis

**Affiliations:** 1 Hematology Laboratory-Blood Bank, Aretaieion Hospital, National and Kapodistrian University of Athens, Athens, Greece; 2 First Internal Medicine Clinic, Laiko Hospital, National and Kapodistrian University of Athens, Athens, Greece; 3 Blood Transfusion Department, General Hospital Sismanoglio, Athens, Greece; 4 Blood Transfusion Department, General Hospital Thriasio, Athens, Greece; 5 2nd Department of Pathology, National and Kapodistrian University of Athens, “Attikon” University Hospital, Athens, Greece; 6 Hellenic National Blood Transfusion Center, Athens, Greece

**Keywords:** Alloimmunization, Prevalence, Red blood cells, Alloantibodies, Specificity, Age distribution, Sex distribution, Blood transfusion

## Abstract

**Objective:**

Blood transfusion is a common lifesaving treatment but it is often complicated with alloimmunization. Previously studies in Greece have concentrated on alloimmunization in multiply transfused thalassemic patients or antenatal women. However, the relative frequency of red blood cell (RBC) alloantibodies in the general patient population has not been studied so far. The aim of the present retrospective study was to estimate the prevalence and specificity of RBC alloantibodies in a large cohort of patients in two general hospitals and their association with age, sex, and the patients’ clinic of hospitalization.

**Materials and Methods:**

Data from 2012 to 2016 from the “Sismanogleio” and “Thriasio” general hospitals in Athens, Greece, were studied retrospectively. Statistical analysis was performed with SAS for Windows 9.4.

**Results:**

Six hundred twenty-six patients (626/53800, 1.16%) were alloimmunized for one or more alloantibodies. The mean age was 67.99±17.56 years. Most antibodies were found in women [62.66% (438/699) in women vs. 37.34% (261/699) in men (p=0.0007)], while the vast majority of antibodies (66.81%) were found in patients aged 61-90. The most frequent antibody was anti-Kell (26.61%), followed by anti-E (16.02%), anti-D (15.02%), anti-Jka (5.87%), and anti-M (5.72%). Anti-C (81.48%, n=27) and anti-Cw (54.17%, n=24) tended to be found more often in patients with multiple antibodies. Most alloimmunized cases were found in general surgery (42.65%) and internal medicine departments (38.66%).

**Conclusion:**

According to our results, the alloimmunization data in a general patient population in Greece were consistent with the majority of studies in the international literature. Whether a strategy at national level needs to be directed towards extending matching for the whole population or towards applying sensitive and compulsory indirect antiglobulin tests before any transfusions in order to efficiently prevent alloimmunization remains an issue of debate.

## Abstract (tr)

**Amaç:**

Kan transfüzyonu sık uygulanan hayat kurtarıcı bir tedavidir ancak sıklıkla alloimmünizasyon ile komplike olur. Yunanistan’da daha önce yapılan çalışmalar çoklu transfüze edilen talasemik hastalar ve antenatal kadınlar üzerine yoğunlaşmıştır. Ancak şimdiye kadar genel hasta popülasyonunda eritrosit alloantikorlarının görece sıklığı araştırılmamıştır. Bu geriye dönük çalışmanın amacı iki hastanedeki büyük bir hasta grubunda eritrosit alloantikorlarının sıklığı, özgüllüğü yanında yaş, cinsiyet ve hastaneye yatış kliniği ile ilişkisi tahmin etmektir.

**Gereç ve Yöntemler:**

Atina, Yunanistan’da bulunan Sismanogleio ve Thriasio hastanelerinin 2012-2016 dönemine ait verileri geriye dönük olarak incelendi. İstatistiksel analiz SAS for Windows 9.4 yazılım platformu ile yapıldı.

**Bulgular:**

Altı yüz yirmi altı hastanın (626/53800, %1,16) bir veya daha fazla alloantikor ile alloimmünize olduğu saptandı. Ortalama yaş 67,99±17,56 idi. Kadınlarda antikor saptanma oranı (%62,66; 438/699) erkeklere (%37,34; 261/699) kıyasla daha fazlaydı (p=0,0007) ve antikorların büyük çoğunluğu (%66,81) 61-90 yaş grubunda izlendi. En sık anti-Kell antikoru (%26,61) saptanırken bunu anti-E (%16,02), anti-D (%15,02), anti-Jka (%5,87) ve anti-M (%5,72) izledi. Anti-C (%81.48, n=27), anti-Cw (%54,17, n=24) çoklu antikorlu hastalarda daha sık bulunma eğilimindeydi. Alloimmünize olgularının çoğu genel cerrahi (%42,65) ve iç hastalıkları (%38,66) kliniğinde bulundu.

**Sonuç:**

Sonuçlarımıza göre, Yunanistan’da bulunan genel hasta popülasyonundaki alloimmünizasyon oranları uluslararası literatürdeki çalışmaların çoğu ile uyumludur. Alloimmünizasyonun etkin biçimde önlenmesi için ulusal düzeydeki stratejinin tüm popülasyonda genişletilmiş uyum arama veya tüm transfüzyonların öncesinde zorunlu indirekt antiglobulin testine yönlendirilmesi konuşu tartışmalıdır.

## Introduction

Blood transfusion is a common and lifesaving treatment but it is often complicated with adverse reactions such as alloimmunization. Alloimmunization occurs because of red cells’ antigenic differences between donor and recipient or between mother and fetus. Alloimmunization is implicated in the pathogenesis of hemolytic reactions (acute or delayed) and hemolytic disease of the fetus and newborn [1,2].

The most important factors that influence alloimmunization are the number of red blood cell (RBC) units transfused, with the risk increasing with increasing number of transfusions and female sex (since women are more susceptible to exposure to alloantigens during pregnancy, miscarriages, abortions, and childbirth), while solid tumors, lymphoproliferative diseases, leukemia, and diabetes mellitus are also factors that may modulate the risk for alloimmunization [3,4,5].

Studies so far have reported various immunization rates among different study groups, ranging from 5% in the general population and a mean percentage of 1.6% in pregnant women worldwide [6,7] to up to 30% in multitransfused patients (myelodysplastic syndromes, thalassemia patients) [8,9]. Genetic heterogeneity between donor and recipient populations, differences in transfusion policies, and differences in the specificity and sensitivity of the test methods may account for the reported variation [10,11,12,13].

Although in Greece there are no official national guidelines, it is common practice for thalassemia patients and women of reproductive age to undergo preemptive antigen matching for Rh (C, c, E, e) and K in order to prevent alloimmunization and improve transfusion safety by reducing alloantibody formation.

Previously performed studies in Greece have focused on multitransfused thalassemic patients or antenatal women [7,14]. However, the relative frequency of RBC alloantibodies in the general patient population has not been studied so far. Accordingly, the aim of the present retrospective study was to estimate the prevalence and specificity of RBC alloantibodies in a large cohort of hospitalized patients and to associate them with their age, sex, and clinic of hospitalization.

## Materials and Methods

### Study Population and Baseline Characteristics

Two general hospitals were selected retrospectively for data collection from the start of 2012 to the end of 2016.

In Greece all patients are phenotyped for ABO, RhD, and CcEe and Kell. All patients receive ABO/RhD-compatible RBCs and, when feasible, CcEe- and Kell-compatible RBCs. A screening test with an indirect antiglobulin test (IAT) for alloantibody detection was performed for all patients. The IAT was performed using the gel microcolumn agglutination technique with two different commercially available systems: i) AutoVue and BioVue (Ortho Clinical Diagnostics, Bridgend, United Kingdom) and/or ii) DiaMed-ID (DiaMed AG, Cressier sur Morat, Switzerland). An alloantibody identification test to identify antibody specificity was performed in every case of a positive screening test by using a panel of 11 commercially available test erythrocytes of known antigenic synthesis (0.8% Resolve Panel C System, Ortho Clinical Diagnostics Systems, Bridgend, United Kingdom and/or ID-DiaPanel/DiaPanel-P Bio-Rad, DiaMed, Cressier sur Morat, Switzerland). In all cases of a positive screening test, age, sex, and the clinic in which the patient was hospitalized were recorded.

### Statistical Analysis

Statistical analysis was performed with SAS for Windows 9.4 (SAS Institute Inc., Cary, NC, USA). Descriptive values were expressed as mean±standard deviation (SD) or percentages within groups. Comparisons between groups for the categorical parameters were performed by Fisher’s exact test, while for dichotomous categorical variables odds ratio analysis was performed. For the arithmetic parameters (such as age or number of antibodies) the Mann-Whitney U test was applied since normality was not possible to be always ensured. The significance level (p-value) was set at <0.05 [15].

## Results

IAT was performed for 53800 patients in both participating hospitals. Six hundred twenty-six patients (626/53800, 1.16%) were found positive for one or more alloantibodies. The mean age of patients in whom an alloantibody was identified was 67.99±17.56 years, ranging from 20 to 98 (median: 73 years). Two hundred thirty-nine of those patients were male (239/626, 38.18%, mean age: 69.38±15.94 years) and 378 were female (378/626, 61.82%, mean age: 67.10±18.46 years). In Table 1 the baseline characteristics of the study population are presented.

### Frequency of Antibodies

The frequency of identified antibodies was calculated by either counting the patients who had the specific antibody and considering multiple occurrences separately or by counting the occurrence of each individual antibody without considering if a patient had multiple antibodies.

The most frequent antibody was anti-Kell (26.61%), followed by anti-E (16.02%), anti-D (15.02%), anti-Jka (5.87%), and anti-M (5.72%).

Of the 626 patients, 556 (88.82%) had a single antibody while 70/626 patients (11.18%) had multiple antibodies. The majority of them had two antibodies, and only three patients had 3 antibodies (4.29% of the population with multiple antibodies and 0.48% of the positive population), and no patient was found to have more than three antibodies. On average, 1.12±0.34 antibodies were found per patient.

Anti-C (81.48%, n=27) and anti-Cw (54.17%, n=24) tended to be more often found in patients with multiple antibodies. The details of the frequency of patients with specific antibodies and the tendency of each antibody for single or multiple occurrences are depicted in Table 2.

### Antibodies and Sex

Most of the antibodies were found in women (62.66%, 438/699) and 37.34% (261/699) were found in men (p=0.0007). The distribution of alloantibodies between the sexes is presented in Table 3 (p<0.0001).

In an effort to investigate whether specific antibodies were more frequent in men or women, we performed Fisher’s exact test for all patients in comparison to each specific antibody (presence or absence), as depicted in Table 3. Notably, in the majority of cases, men had lower odds to develop an antibody than women (odds ratio <1).

Anti-D and anti-C were more likely to be found in women than men (in all cases p<0.05). Moreover, all anti-Fyb-positive individuals (n=7) were female and all anti-s (n=3) individuals were male (in both cases p<0.05) (Table 3).

Multiple antibodies were identified in 70/626 cases. Forty-nine of 387 women (12.66%) and 21 of 239 men (8.79%) developed multiple antibodies (p=0.1350) (see Table A1 in the Appendix).

### Antibodies in Different Age Groups

The population was separated into age groups by 5- and 10-year intervals and the frequency of the antibodies was investigated. A difference in the distribution of each individual antibody between some age groups (Fisher exact test, p<0.0001 for 5-year intervals and p<0.0001 for 10-year intervals) was found. No statistical significance in age between males and females was found (median age for males: 75 years, q1-q3: 60-81 years, for women: 72 years, q1-q3: 56-81 years, p=0.2363).

The majority of antibodies (30.9%) were found in the age group of 71-80 years, and in general the vast majority of antibodies (15.31%+30.9%+20.6%=66.81%) were found in individuals aged 61-90 years. A detailed distribution of the antibodies according to different age groups is shown in Table A2 and Table A3 in the Appendix.

**Figure 1:**
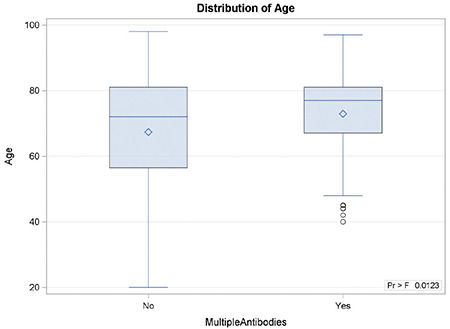
Box plot for the distribution of patient age by multiple antibodies.

When we compared patients’ age with multiple antibodies with patients’ age with single antibodies (see Figure 1), it was found that the median age of patients with multiple antibodies (n=70) was 77 (q1-q3: 67-81 years) and the median age of patients with a single antibody (n=556) was 72 years (q1-q3: 56.5-81 years) (p=0.0180).

From 50 to 100 years, in both men and women, the most common alloantibody was anti-Kell (158/607, 26.03%). This was followed by anti-E (96/607, 15.82%) and then by anti-D (78/607, 12.85%).

### Antibodies by Clinic

The majority of cases with a positive IAT were found in general surgery (42.65%) and in internal medicine departments (38.66%). Anti-Kell was the most frequent antibody in all the departments (more details of the distribution of antibodies in the clinics are presented in Table A4 in the Appendix).

## Discussion

The development of anti-erythrocytic antibodies (allo- and autoantibodies) affects multitransfused patients to varying degrees and can significantly complicate transfusion. The development of unexpected, clinically important antibodies is associated with an increased risk of acute and delayed hemolytic reactions following transfusion, as well as hemolytic disease in neonates. Furthermore, the crossmatch incompatibility that the development of anti-erythrocyte antibodies may cause is a potentially complex problem with an impact on blood availability in urgent medical situations.

In Greece, to date, the data on alloimmunization in the general patient population have been poor, and most studies were performed on multitransfused patients, mainly those with thalassemic syndromes, multiparous women, and patients who received chemotherapy for solid organ tumors and hematological malignancies. Our study is the first one conducted on a large scale within the Greek general patient population, as neither of the participating hospitals have thalassemia or obstetrics units. 

The limitation of our study was the lack of data on the transfusion and/or pregnancy history of our patients and we thus could not report the time of development of an alloantibody (i.e., whether patients had previously had a positive IAT).

In a total of 53800 cases screened, the alloimmunization rate was 1.16%, a rate similar to those of other studies from the general population of patients that reported a rate of alloimmunization ranging from 0.46% to 2.4% [16,17,18,19,20].

The prevalence of alloimmunization is much lower than that reported for multitransfused patients, especially those with hemoglobinopathies, which ranges from 8% up to 56%, especially in cases of sickle cell disease [16,17,18,19,20,21,22,23,24,25].

In a recent study in Greece with patients with hemoglobinopathies the prevalence of alloimmunization up to 2010 was 11.6%, while after 2010, when an extended matching strategy was applied (including ABO, CcDEe, and Kell), the alloimmunization rate decreased to 1.4%, similar to the rate recorded in our study for the general population [21].

### Specificity of Alloantibodies

In the study hospitals, apart from ABO/RhD typing, extended Rhesus phenotyping (CcEe) and K typing were also performed. All patients receive ABO/RhD-compatible RBCs and, when feasible, CcEe- and Kell-compatible RBCs. This may have affected the rates of alloimmunization as well as alloantibody specificities. The most frequent antibody was anti-Kell (26.61%), followed by anti-E (16.02%), anti-D (15.02%), anti-Jka (5.87%), and anti-M (5.72%), a finding similar to that reported by other studies of Greek populations [7].

The outcome of our study is also consistent with many studies in the American general patient population, which have shown that anti-Kell is the most common alloantibody [16,26,27]. On the contrary, other studies from France and Germany have shown that anti-E was the most common alloantibody [20,28,29]. Differences in alloantibodies’ specificities may arise from the different methodologies applied among different blood bank establishments. IAT methods that enhance the detection of Rhesus alloantibodies (i.e., performing an additional IAT with papain cells) can result in detecting anti-E more frequently when compared to other methods that do not enhance Rhesus alloantibodies, such as albumin [30].

### Coexistence of Alloantibodies

Of the 626 patients, 556 (88.82%) had a single antibody while 70/626 patients (11.18%) had multiple antibodies. Double alloantibodies were detected in 10.72% of the patient population with the more frequent combination being anti-(D + C), a finding that was similar to results reported from other studies that showed that combinations against Rhesus and Kell antigens were the most frequent [31,32,33].

### Sex and Age

In our study, 261 alloantibodies were found in males (37.34%) and 438 in females (62.66%). The male/female ratio was approximately 1:1.7. The rate of alloimmunization found in our study according to sex is similar to those of other studies that reported male/female ratios ranging from 1.8 to 2.7 [26,29,31,34], although there are also studies that have shown no difference in the rates of sensitization between men and women [33,34,35]. Considering that the common practice applied in Greece is to test RhD-negative blood donor samples for weak/partial D by IAT before the final release of RhD-negative RBC units, the case of sensitization due to RBC unit transfusions with weak expression of RhD that had been mistakenly identified as RhD-negative is highly unlikely.

The higher rates of alloimmunization reported in women may be due, in part, to their longer life expectancy, but also to their antigenic exposure during pregnancy, as opposed to transfusions being the only source of exposure in men. Embryo-fetal manipulation (EFM) is a common physiological phenomenon that persists for decades after pregnancy [36]. Embryonic-derived semi-allogenic functional T, B, and NK lymphocytes and monocytes have been detected in the blood circulation in women with past pregnancies [37]. It is likely that EFM provides female blood recipients with a second immune system that can act primarily on exposure to transfused alloantigens and increase the risk of generating anti-erythrocyte antibodies during their whole lives. In our study, we noticed that alloimmunization rates in women gradually increased from 50 years onwards compared to male patients [38].

When the population of our survey was separated into age groups (either by 5 years or 10 years) there was a difference in the distribution of each individual antibody into the age groups (p<0.0001 both for 5-year and 10-year intervals). For the first time in the literature, we performed an analysis of the age distribution of alloantibodies in an age range from 20 to 100 years and the majority of antibodies (30.9%) were found in the age group of 71-80 years. The vast majority of antibodies (15.31%+30.9%+20.6%=66.81%) were found in patients aged 51-100 years. However, since the distribution of the examined population in the age groups is not known, it is not possible to estimate the frequency of antibodies in the general population. It is also quite important that as we move up the decades from 50 to 100 years, in both men and women, the most common alloantibody that emerges is anti-Kell, followed by anti-E and then by anti-D.

It was also found that patients with multiple antibodies were older in comparison to patients with a single antibody (p=0.0180; median: 77 years vs. 72 years). These findings indicate that either the patients were exposed in the past to RBC transfusions not matched for Rh subgroup CcEe and K phenotype, or that laboratory service practices tend not to be strict about transfusing extended antigen-matched blood in older patients.

Regarding the distribution of alloantibodies by hospital department, the majority of antibodies were found in internal medicine (39.20%) and general surgery (42.35%), probably due to the fact that most patients in general hospitals, and especially the elderly, are treated in these departments. Notably, anti-Kell was the most frequent antibody in all the departments.

## Conclusion

In this 5-year retrospective study, we assessed the frequency of alloantibodies in a Greek population of patients, as well as their associations with different age groups, the sex of the patients, and the clinic of hospitalization. All our findings were consistent with the majority of studies in the international literature. Concluding, we can state that the most commonly found alloantibodies belong to the Rhesus and Kell systems and that women tend to develop multiple antibodies.

For multitransfused patients, alloimmunization is associated with major Rh and Kell antigens. Further extended typing including MNS, Duffy, Kidd, and other immunogenic antigens is considered to be especially important to reduce alloantibody formation, to avoid hemolysis transfusion reactions, and to improve transfusion safety.

Applying this practice to all transfusion recipients, although ideal, could be costly and practically not feasible. Whether a strategy at national level has to be directed towards extending matching for the whole population or towards applying sensitive and compulsory IAT before any transfusion in order to efficiently prevent alloimmunization remains an issue of debate.

## Figures and Tables

**Table 1:**
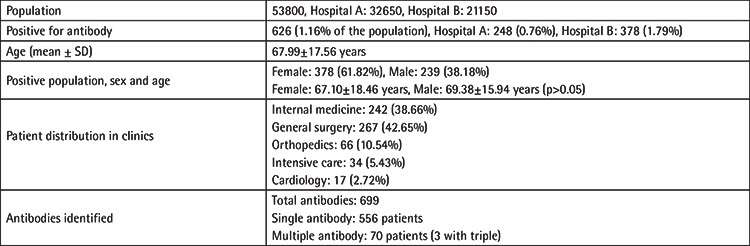
Baseline characteristics of the study population.

**Table 2:**
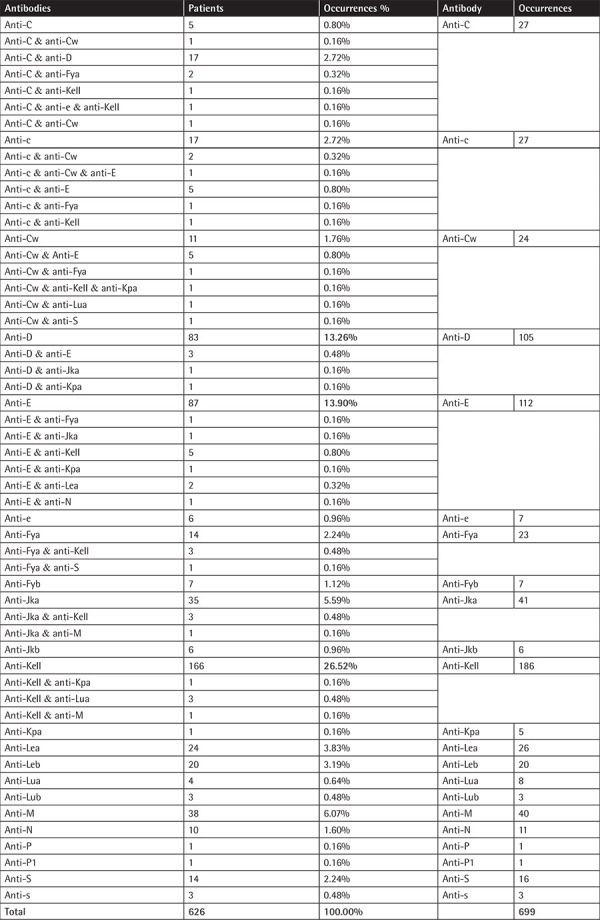
Number of patients with each specific antibody and occurrences of every antibody identified along with single and multiple occurrence percentages (bold values indicate comparisons with a statistically significant difference).

**Table 3:**
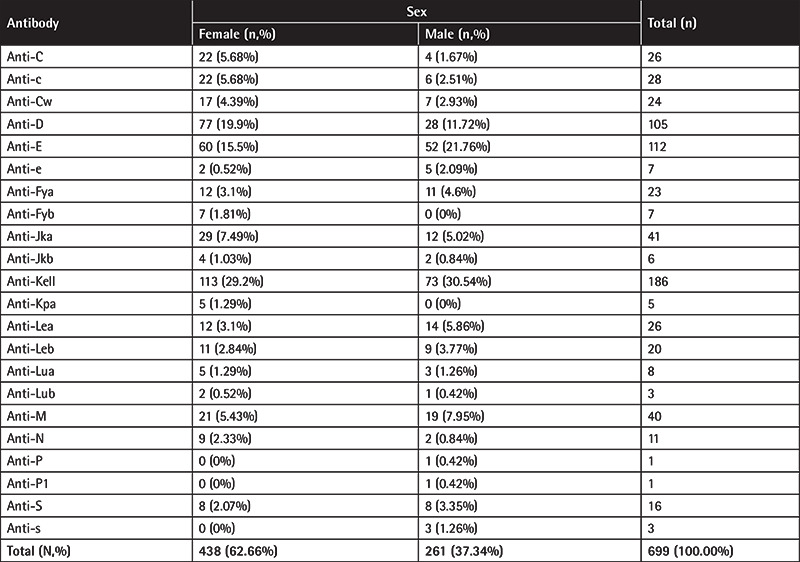
Antibodies’ specificities by sex. For each sex and individual antibody’s specificity, the number of antibodies identified and the percentage of females and males that developed each antibody are reported.

**Table A1:**
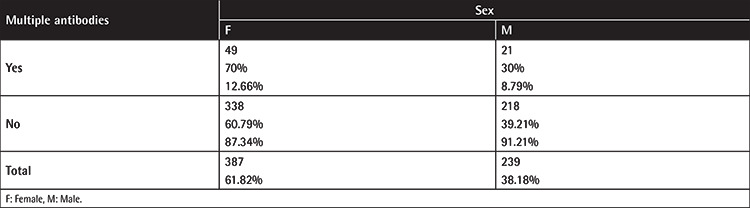
Single vs. multiple antibodies by sex, each box indicating the number of antibodies, the row’s percentage, and the column’s percentage.

**Table A2:**
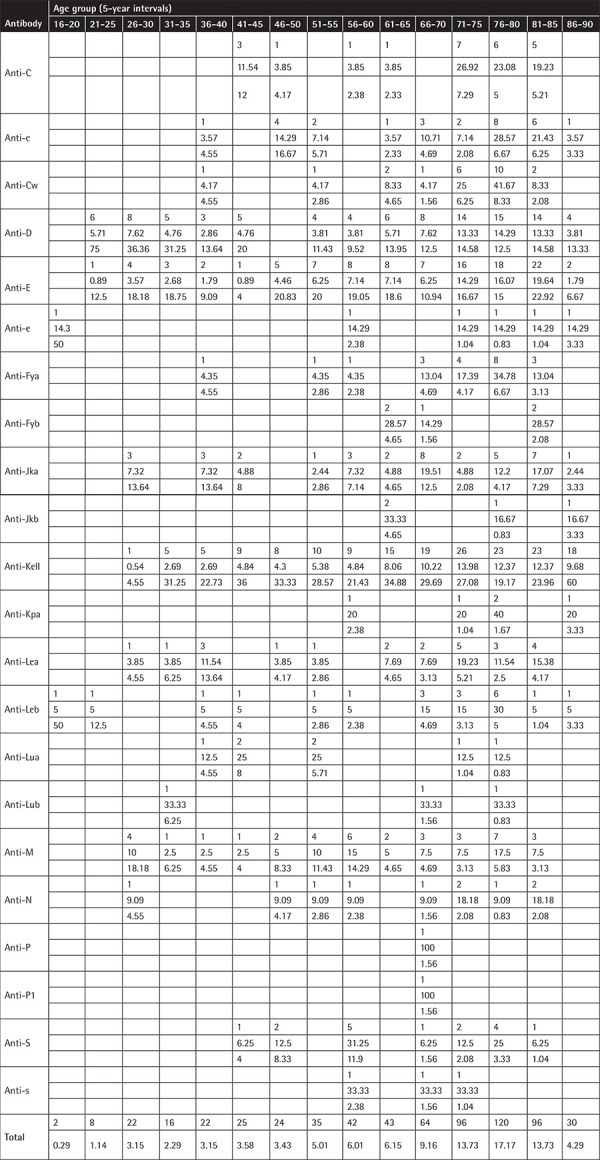
Antibodies by age groups using 5-year intervals (for each antibody and age group the number of antibodies, the row’s percentage, and the column’s percentage are displayed).

**Table A3:**
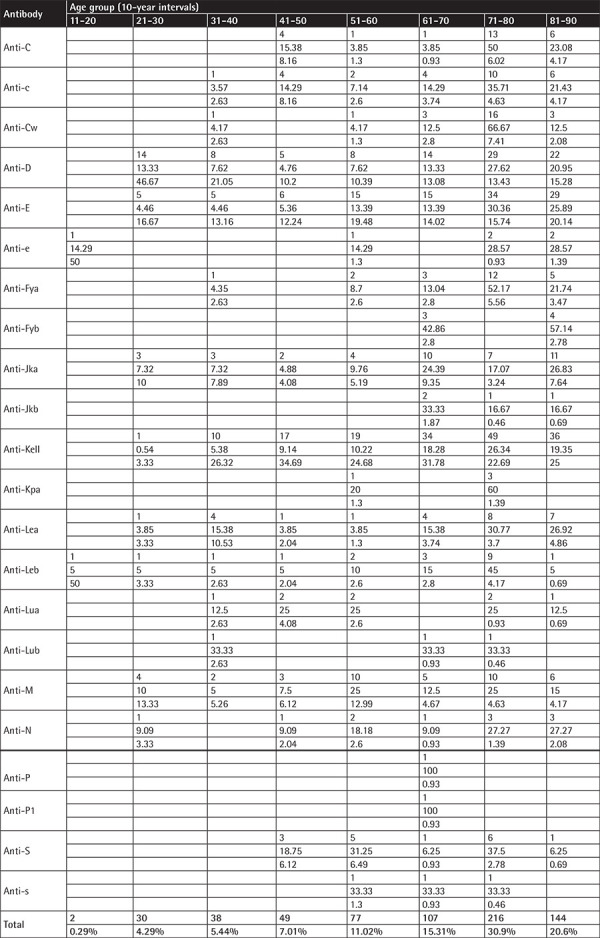
Antibodies by age groups using 10-year intervals (for each antibody and age group the number of antibodies, the row’s percentage, and the column’s percentage are displayed).

**Table A4:**
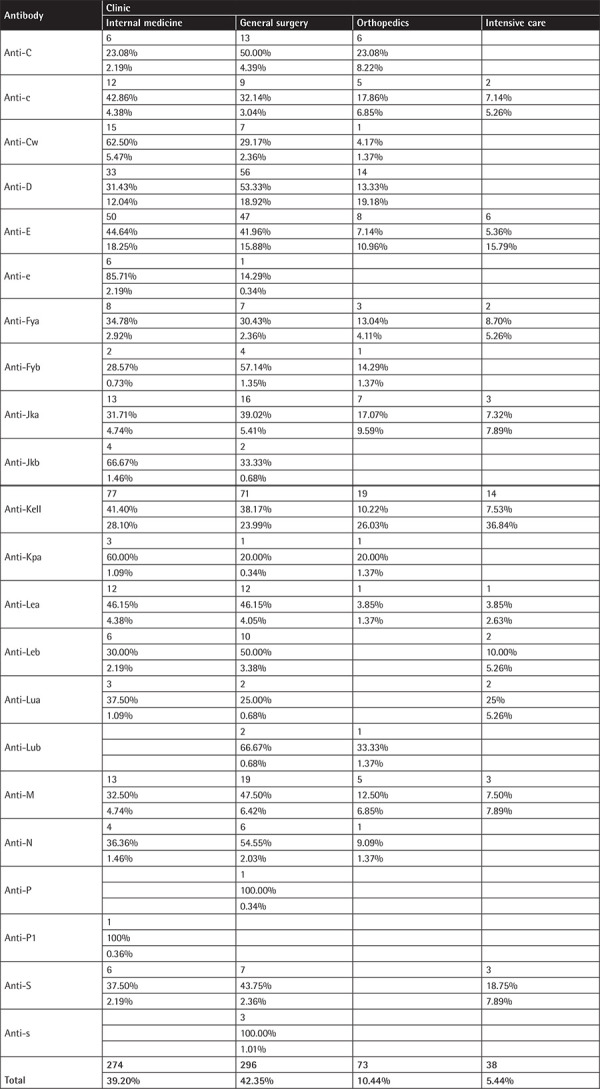
Antibodies by clinic (for each antibody and clinic the number of antibodies, the percentage of the specific antibody in the clinic (row percentage), and the percentages of all antibodies in the specified clinic (column percentage) are displayed). Bold values indicate comparisons with a statistically significant difference.

## References

[1] Schonewille H (2008.). Red Blood Cell Alloantibodies after Transfusion. Leiden, Leiden University Press.

[2] de Haas M, Thurik FF, Koelewijn JM (2015). Haemolytic disease of the fetus and newborn. Vox Sang.

[3] Petz LD, Garratty G (2004.). Immune Hemolytic Anemia. 2nd ed. Philadelphia, Churchill-Livingstone.

[4] Zalpuri S, Zwaginga JJ, le Cessie S, Elshuis J, Schonewille H, van der Bom JG (2012). Red-blood-cell alloimmunization and number of red-blood-cell transfusions. Vox Sang.

[5] Hoeltge GA, Domen RE, Rybicki LA, Schaffer PA (1995). Multiple red cell transfusions and alloimmunization. Experience with 6996 antibodies detected in a total of 159,262 patients from 1985 to 1993. Arch Pathol Lab Med.

[6] Velkova E (2015). Correlation between the amount of anti-D antibodies and IgG subclasses with severity of haemolytic disease of foetus and newborn. Open Access Maced J Med Sci.

[7] Foudoulaki-Paparizos L, Valsami S, Bournas N, Tsantes A, Grapsas P, Mantzios G, Travlou A, Politou M (2013). Alloimmunisation during pregnancy in Greece: need for nationwide HDFN prevention programme. Transfus Med.

[8] Seyfried H, Walewska I (1990). Analysis of immune response to red blood cell antigens in multitransfused patients with different diseases. Mater Med Pol.

[9] Fluit CR, Kunst VA, Drenthe-Schonk AM (1990). Incidence of red cell antibodies after multiple blood transfusion. Transfusion.

[10] Ramsey G, Hahn LF, Cornell FW, Boczkowski DJ, Staschak S, Clark R, Hardesty RL, Griffith BP, Starzl TE (1989). Low rate of Rhesus immunization from Rh-incompatible blood transfusions during liver and heart transplant surgery. Transplantation.

[11] Shukla JS, Chaudhary RK (1999). Red cell alloimmunization in multi-transfused chronic renal failure patients undergoing hemodialysis. Indian J Pathol Microbiol.

[12] Reisner EG, Kostyu DD, Phillips G, Walker C, Dawson DV (1987). Alloantibody responses in multiply transfused sickle cell patients. Tissue Antigens.

[13] Hendrickson JE, Tormey CA (2016). Red blood cell antibodies in hematology/oncology patient interpretation of immunohematology. Test and clinical significance of detected antibodies. Hematol Oncol Clin.

[14] Petrakos G, Andriopoulos P, Tsironi M (2016). Pregnancy in women with thalassemia: challenges and solutions. Int J Womens Health.

[15] DiMaggio C (2014.). SAS for Epidemiologists: Applications and Methods. New York, Springer.

[16] Tormey CA, Fisk J, Stack G (2008). Red blood cell alloantibody frequency, specificity and properties in a population of male military veterans. Transfusion.

[17] Ko KH, Yoo BH, Kim KM, Lee WY, Yon JH, Hong KH, Han TH (2012). Frequency of unexpected antibody and consideration during transfusion. Korean J Anesthesiol.

[18] Chaudhary R, Agarwal N (2011). Safety of type and screen method compared to conventional antiglobulin crossmatch procedures for compatibility testing in Indian setting. Asian J Transf Sci.

[19] Lee WH, Kim SY, Kim HO (2000). The incidence of unexpected antibodies in transfusion candidates. Korean J Transf.

[20] Shin JH, Lee JY, Kim JH, Kim HR, Lee JN (2009). Screening and identification of unexpected red cell antibodies by simultaneous Liss/Coombs and NaCl enzyme gel methods. J Korean Med Sci.

[21] Politis C, Hassapopoulou E, Halkia P, Kourakli A, Mougiou A, Zervou E, Kleronomos E, Sfyridaki K, Pappa C, Tsoumari I, Lafiatis I, Kavallierou L, Parara M, Richardson C (2016). Managing the patient with haemoglobinopathy and multiple red cell antibodies. ISBT Science Series.

[22] Chao YH, Wu KH, Lu JJ, Shih MC, Peng CT, Chang CW (2013). Red blood cell alloimmunization among Chinese patients with α-thalassaemia major in Taiwan. Blood Transfus.

[23] Cheng CK, Lee CK, Lin CK (2012). Clinically significant red blood cell antibodies in chronically transfused patients: a survey of Chinese thalassemia major patients and literature rev. Transfusion.

[24] Treml A, King KE (2013). Red blood cell alloimmunization: lesson from sickle cell disease. Transfusion.

[25] Woldie I, Swerdlow P, Bluth MH, Mohammad U, Landolfi E, Chaudrhy S, Dyson G, O’Malley BA (2015). Lifetime risk and characterization of red blood cell alloimmunization in chronically transfused patients with sickle cell disease. J Blood Transfus Immunohematol.

[26] Walker RH, Lin DT, Hartrick MB (1989). Alloimmunization following blood transfusion. Arch Pathol Lab Med.

[27] Fluit CRMG, Kunst VAJM, Drenthe-Schonk AM (1990). Incidence of red cell antibodies after multiple blood transfusion. Transfusion.

[28] Spielmann W, Seidl S (1974). Prevalence of irregular red cell antibodies and their significance in blood transfusion and antenatal care. Vox Sang.

[29] Duboeuf S, Flourié F, Courbil R, Benamara A, Rigal E, Cognasse F, Garraud O (2012). Identification of alloantibodies and their associations: Balance sheet of a year at the Auvergne-Loire French Blood Establishment. Transfus Clin Biol.

[30] Winters JL, Pineda AA, Gorden LD, Bryant SC, Melton LJ 3rd, Vamvakas EC, Moore SB (2001). RBC alloantibody specificity and antigen potency in Olmsted County, Minnesota. Transfusion.

[31] Achargui S, Zidouh A, Abirou S, Merhfour FZ, Monsif S, Amahrouch S, El Ghobre A, El Halhali M, Temmara H, El Hryfy A, Motqi M, Satty A, Kandili M, Aghri M, Hajjout K, Benajiba M (2017). Identification of alloantibodies and their associations: balance sheet of 3 years at the Regional Center of Blood Transfusion in Rabat/Morocco and difficult in transfusion management. Transfus Clin Biol.

[32] Azarkeivan A, Ahmadi MH, Zolfaghari S, Shaiegan M, Ferdowsi S, Rezaei N, Lotfi P (2015). RBC alloimmunization and double alloantibodies in thalassemic patients. Hematology.

[33] Giblett ER (1977). Blood group alloantibodies: an assessment of some laboratory practices. Transfusion.

[34] Murao M, Viana MB (2005). Risk factors for alloimmunization by patient with sickle cell disease. Braz J Med Biol Res.

[35] Spanos T, Karageorga M, Ladis V, Peristeri J, Hatziliami A, Kattamis C (1990). Red cell alloantibodies in patients with thalassemia. Vox Sang.

[36] Blumberg N, Ross K, Avila E, Peck K (1984). Should chronic transfusions be matched for antigens other than ABO and Rho(D)?. Vox Sang.

[37] Verduin EP, Brand A, Middelburg RA, Schonewille H (2015). Female sex of older patients is an independent risk factor for red blood cell alloimmunization after transfusion. Transfusion.

[38] Khosrotehrani K, Johnson KL, Cha DH, Salomon RN, Bianchi DW (2004). Transfer of fetal cells with multilineage potential to maternal tissue. JAMA.

